# Effects of Sequential Combination of Moderate Pressure and Ultrasound on Subsequent Thermal Pasteurization of Liquid Whole Egg

**DOI:** 10.3390/foods12132459

**Published:** 2023-06-23

**Authors:** Ana C. Ribeiro, Susana Casal, José A. Lopes da Silva, Jorge A. Saraiva

**Affiliations:** 1 Associate Laboratory LAQV-REQUIMTE, Department of Chemistry, University of Aveiro, 3810-193 Aveiro, Portugal; 2 Associate Laboratory LAQV-REQUIMTE, Laboratory of Bromatology and Hydrology, Faculty of Pharmacy, Department of Chemistry, University of Porto, 4050-313 Porto, Portugal

**Keywords:** egg products properties, moderate pressure, ultrasound, thermal pasteurization, *Salmonella*

## Abstract

As an alternative to commercial whole egg thermal pasteurization (TP), the sequential combination of moderate pressure (MP) and/or ultrasound (US) pre-treatments prior to a shorter TP was evaluated. The use of US alone or in combination with MP or TP resulted in an inactivation that was far from that of commercial TP. Nevertheless, when these three technologies were combined (MP–US–TP, 160 MPa/5 min–50% amplitude/1 min–60 °C/1.75 min), a safety level comparable to that of commercial TP was established. This was likely due to a decrease in the thermal resistance of *Salmonella* Senftenberg 775/W caused by MP and US pre-treatments. Regarding liquid whole egg (LWE) properties, using raw LWE as a reference, TP and MP treatments each decreased protein solubility (7–12%), which was accompanied by a viscosity increment (41–59%), whereas the US-only and MP–US–TP treatments improved protein solubility (about 4%) and reduced viscosity (about 34%). On average, all treatments lowered the emulsifying properties of LWE by 35–63%, with the MP–US–TP treatment having a more dramatic impact than commercial TP. In addition, the US-only, MP-only, and MP–US–TP treatments had the greatest impact on the volatile profile of LWE, lowering the concentration of the total volatile components. In comparison to commercial TP, LWE treated with MP–US–TP exhibited greater protein solubility (19%), lower viscosity (56%), and comparable emulsifying stability, but with a decreased emulsifying capacity (39%) and a lower total volatile compounds content (77%). Considering that a combined treatment (MP–US–TP) is lethally equivalent to commercial TP, but the latter better retained the quality properties of raw LWE, including volatiles, the application of MP followed by US pre-treatments before a shorter TP did not demonstrate significant advantages on quality parameters in comparison to commercial TP.

## 1. Introduction

Salmonellosis is the second-most-frequent human zoonosis in the European Union, despite a notable downward trend since 2008 [1]. The most prevalent serovars are *Salmonella* Enteritidis and *S.* Typhimurium, and eggs and egg products continue to be the food vehicles that are most frequently associated with human infection, being associated with 44.0% of strong-evidence *Salmonella* outbreaks in 2020 [1,2].

Eggs are a nutritious part of our diet and a useful ingredient in foods, due to their functional properties [3], but pasteurization is required for the easy use of liquid egg products as a replacement for shell eggs in the food business [4]. Accordingly, egg products’ thermal pasteurization (TP) has been intended largely to prevent *Salmonella* spp. TP may be administered at 55.6–70 °C for 1.5–6.2 min, depending on the egg product [5,6,7]. Nevertheless, heat treatments designed to achieve this safety level can decrease soluble protein, foaming, and emulsifying properties, thus hindering eggs’ use as food ingredients [8]. Therefore, new strategies are under research with the aim of obtaining *Salmonella*-free egg products with reduced impact on eggs’ quality properties.

To ensure the safety of egg products, non-thermal technologies such as high pressure (HP, 300–450 MPa for 10–30 min) and ultrasound (US, 24.6–42 W, 1–30 min, <30 °C) have been studied. However, their use per se was shown to be ineffective in reducing a 5 log_10_ colony forming unit (CFU)/g of a foodborne pathogen, and they also had a significant negative impact on eggs’ quality by causing possible interesting microbial inactivation; therefore, they are not viable alternatives [9,10,11,12,13,14,15].

One strategy, developed by Leistner (1995) [16], consists of a combination of different preservation techniques, while lowering their individual intensity in order to avoid quality losses and to achieve a higher impact in controlling microbial growth [17]. For instance, the sequential combination of US (40 W/50 °C/3–5 min, 24.6–42.0 W/5 min) followed by pressure (138 MPa/4–8 min; 250–300 MPa/3–15 min/5 °C) reduced ≤3 log_10_ cycles of *Escherichia coli* and the *S.* Enteritidis population in liquid whole egg (LWE); however, *Listeria innocua* was inactivated in only <0.5 log_10_ cycles. Changing the order of treatments (pressure before US) marginally enhanced *S.* Enteritidis inactivation, although the combination of pressure or US with pulsed electric fields did not enhance microorganisms’ inactivation [18,19]. Nonetheless, the properties of LWE treated by US prior to a pressure treatment exhibited enhanced foaming capacity, probably due to the homogenization effect of US, with no significant change in foaming stability compared to that of non-treated samples [20]. However, none of these treatments were feasible alternatives for achieving the same inactivation level of commercial TP (70 °C/1.5 min), which consists of about 3.5–5.0 log_10_ cycles of *S.* Senftenberg 775 W and *L. monocytogenes* [8].

The application of a pre-treatment (moderate pressure (MP) and/or US) at sublethal intensities, causing sublethal damages in microorganisms and, thereby, inducing greater microorganism sensitivity to a subsequent less-intense TP, is a possible strategy for improving egg-product pasteurization using combined treatments. Therefore, in this work, a combined process using TP at a commercial pasteurization-like temperature (60 °C), during a shorter pasteurization time (1.75 min), preceded by pre-treatments of MP and/or US, was studied in LWE. The primary objective of this study was to evaluate the lethal efficacy of MP (50–160 MPa/5 min) and US (50% amplitude/1–3 min) pre-treatments, alone or in combination, prior to shorter TP (conditions optimization) and, then, to assess the effects on selected egg products’ properties. The lethality of the treatments was evaluated on *S. enterica* serovar Senftenberg, which was selected due to its high heat resistance compared to that of the most frequent serovars (*S.* Enteritidis and *S.* Typhimurium) presented in eggs [2,21]. As far as we are aware, this is the first study to use a sequential combination of three preservation techniques to egg products’ TP. The present study is, accordingly, the first to apply MP and US pre-treatments, before a shorter TP, to LWE, as an attempt to achieve the same microbial lethal effect but with superior quality. Furthermore, unlike other published studies on this topic, the present work did not use chemical additives, in an attempt to meet consumers’ demands for food that is free of chemical additives, and we studied a diversity of parameters with regard to the quality properties of LWE.

## 2. Materials and Methods

### 2.1. Sample Preparation

Fresh shell eggs were obtained from a local supermarket the day before they were used, and held overnight under refrigeration. The eggshells were thoroughly washed with 70% ethanol, allowed to dry at room temperature, then broken aseptically; the whole egg was carefully passed through a colander, with the vitellin membrane disrupted with a scalpel blade, collected in a sterile cup, and gently homogenized for 20 min at 1250 rpm using a magnetic stirrer (MS-3000, Biosan, Rīga, Latvia).

### 2.2. Samples Inoculation and Packaging

*Salmonella enterica* serovar Senftenberg (ATCC 43845) was used in this work (throughout this paper it will be referred to as *S.* Senftenberg 775/W), and a microorganism suspension was prepared, as we previously reported [22]. The frozen suspension was defrosted at room temperature, promptly inoculated into LWE, and mixed for 5 min at 1250 rpm with magnetic stirring (MS-3000, Biosan, Riga, Latvia). Thereafter, non-inoculated and inoculated samples were packed under aseptic conditions in polyamide-polyethylene bags (Plásticos Macar Lda., Santo Tirso, Portugal) that were previously sterilized with ultraviolet radiation and manually thermally sealed to minimize the amount of air inside the bags. The initial concentrations of *S.* Senftenberg 775/W were approximately 10^6^–10^7^ CFU/mL.

### 2.3. Moderate Pressure, Ultrasound and Thermal Pasteurization

The MP treatments were carried out in high-pressure industrial equipment (Model 55, Hyperbaric, Burgos, Spain) with a 55 L pressure vessel, at 20 °C, using water as the pressure-transmitting medium. The US experiments were conducted using lab-scale equipment UP400St (Hielscher Ultrasound Technology GmbH, Teltow, Germany) with a working frequency of 24 kHz and equipped with a 3 mm diameter probe (amplitude at 100% of 166 µm). The probe was used to sonicate 50 mL of LWE in a 100 mL glass beaker, which was maintained at 1.5 cm under the surface of LWE, and the beaker was maintained in an ice-water bath. The sample temperature was measured using a K-type thermocouple, showing an initial temperature of 15 °C, and achieving a maximum temperature of 29 °C. The US treatments were performed in a beaker without any packaging in a biosafety cabinet (Telstar, Bio II Advanced, Barcarena, Portugal). Then, the samples were packaged in polyethylene plastic bags for further processing. For TP, the samples were placed in a circulating water bath (Circulator Bath, FALC, Treviglio, Italy) with the temperature monitored by using a K-type thermocouple (Thermometer 305, Roline, Rolle, Switzerland), and the time was counted after a come-up time of 40 s (previously verified at the geometrical center of the bags (4 × 3 × 0.5 cm)). The combined treatments (MP–TP, US–TP, MP–US, US–MP, MP–US–TP, and US–MP–TP) were performed according to the aforementioned procedures. After treatments, all sample bags were immediately placed on ice and kept at 4 °C until analysis. Each treatment was conducted in triplicate.

Based on pre-tests and previous works, we selected a starting combination of a US treatment (50% amplitude/1 min) with a MP treatment (50 MPa/5 min), followed by a shorter TP (60 °C/1.75 min). Subsequently, based on the results obtained and compared with commercial TP (60 °C/3.5 min, [5,6]), we decided to optimize the processing conditions by varying the US treatment time (50% amplitude/1, 2, and 3 min) and/or the MP intensity (50–160 MPa/5 min).

### 2.4. Microbiological Analysis: Determination of Viable Cell Counts

The samples were analyzed in triplicate for *S.* Senftenberg 775/W counts. After the experiments, 1.0 mL of each sample was collected aseptically and thoroughly mixed with 9.0 mL of Ringer’s solution. Further, decimal dilutions were made with the same diluent, duplicates of the dilutions were plated on Tryptic Soy Agar (TSA, VWR, Carnaxide, Portugal), and the plates were incubated at 37 °C for 24 h. Plates containing 10 to 300 colonies were selected for counting, and the results were expressed as log CFU/mL (microbial load). The results were shown as microbial log load variation (log (N/N_0_)), calculated by the log load difference between the microbial load at each treatment (N) and the initial microbial load (without any treatment) (N_0_) [22,23].

### 2.5. pH and Color

The pH was measured at 20 °C with a properly calibrated glass electrode (pH electrode 50 14, Crison Instruments, S. A., Alella, Spain), by directly submerging it into the homogenized egg samples [22,23].

The color of the LWE samples was evaluated using a Konica Minolta CM 2300 d spectrophotometer (Konica Minolta, Tokyo, Japan), and the color parameters L*–lightness (0, dark; 100, light), a*–redness (+, red;–green), and b*–yellowness (+, yellow;–blue) were obtained, as previously described [22]. The total color difference (ΔE*) was calculated by Equation (1) [24]:

(1) $$\Delta E^{*}={\left[{\left(L^{*}-L_{0}^{*}\right)}^{2}+{\left(a^{*}-a_{0}^{*}\right)}^{2}+{\left(b^{*}-b_{0}^{*}\right)}^{2}\right]}^{1/2}$$

where ∆E* is the total color difference between the sample and the control (raw sample); L* and *L*_0_* represent the lightness of treated and raw samples, respectively; a* and *a*_0_* represent the redness of treated and raw samples, respectively; and b* and *b*_0_* represent the yellowness of treated and raw samples, respectively.

### 2.6. Protein Solubility

The LWE samples were diluted with distilled water to a concentration of 10% (*m*/*v*), then centrifuged at 10,000× *g* during 15 min at 4 °C (Heraeus Biofuge Stratos, Thermo Electron Corporation, Waltham, MA, USA) [15]. The soluble protein content of the supernatant was determined using the microassay Bradford method [25]. A standard curve was obtained using bovine serum albumin (Sigma–Aldrich, Lisbon, Portugal) in the range of 0.02–0.40 g/L. The measurements were conducted in triplicate and the results were expressed in percentages (%) with respect to the values of the non-treated samples.

### 2.7. Thermal Properties

The thermal properties were determined by differential scanning calorimetry (DSC). Egg samples were accurately weighed (7 − 12 ± 2.1 mg) in a stainless-steel pan, then sealed hermetically. A sealed empty pan was used as a reference and the heating rate of the DSC scan was 10 °C/min over a range of 30 to 120 °C [26]. The obtained thermograms were used to determine the peak denaturation temperatures (T_peak_) and enthalpy variation (∆*H*). The DSC measurements were performed in duplicate, and the data were analyzed with the Pyris Series–Diamond DSC Software version 6.0 (PerkinElmer, Waltham, MA, USA).

### 2.8. Apparent Viscosity

As previously described [23], viscosity was measured in a rotational rheometer (Kinexus PRO, Malvern Panalytical, Malvern, UK) with an attached cone-and-plate geometry (a stainless steel cone, 4°, and a diameter of 40 mm). The apparent viscosity of egg samples measured at an intermediate shear rate of 50 s^−1^ was used for comparison among the samples. Measurements were made in triplicate for the samples and in duplicate for analysis.

### 2.9. Emulsifying Properties

The emulsifying activity index (EAI) and the emulsion stability index (ESI) were measured at room temperature, as previously described [23]. The measurements were performed in triplicate for samples and in duplicate for analysis, and the EAI and ESI were calculated by Equations (2) and (3) [27]:

(2) $$\mathrm{EAI}\;\left(m^{2}/g\right)=\frac{2\times 2.303\times A_{0}\times \mathrm{DF}}{c\times \Phi \times 10^{4}}$$

(3) $$\mathrm{ESI}\;\left(\min\right)=\frac{A_{0}}{(A_{0}-A_{10})\times \Delta t}$$

where A_0_ and A_10_ are the absorbance of the diluted emulsions at 0 and 10 min, respectively; DF is the dilution factor; c is the initial concentration of protein (g/mL); Φ is the fraction of oil used to form the emulsion (0.4); and ∆*t* is the time interval between taking the first and second aliquots of emulsion.

### 2.10. Secondary Lipid Oxidation

As previously stated [23], lipid oxidation was evaluated by quantification of secondary lipid oxidation products using the thiobarbituric acid-reactive substances (TBARS) method. A standard curve of malondialdehyde (MDA) was obtained with 1,1,3,3-tetramethoxypropane, in the range of 0.5–10 µM MDA. The measurements were performed in triplicate and the results were expressed as µg MDA/100 g of whole egg.

### 2.11. Volatile Profile

The volatile compounds of LWE samples were extracted using headspace solid-phase microextraction (HS-SPME) and analyzed by gas chromatography-mass spectrometry (GC-MS), as previously reported [23], with minor modifications. In brief, a 2.0 g of LWE in a 15 mL glass vial were added 0.60 g of sodium chloride and 50 µL of the internal standard and immediately sealed with a screw cap with a silicone septum; then, the samples were equilibrated for 15 min at 45 °C followed by HS-SPME exposure at the same temperature under moderate stirring (100 rpm) for 20 min [28]. Semi-quantification was performed using cyclohexanone as the internal standard and the results were expressed in µg internal standard equivalents/100 g of whole egg, with a detection limit of 0.01 µg/100 g of whole egg.

### 2.12. Statistical Analysis

#### 2.12.1. Analysis of Variance

All data were tested at a 0.05 probability level (*p* < 0.05) and the effect of each condition was tested with a one-way analysis of variance (ANOVA), followed by a multiple comparisons test (Tukey’s Honestly Significant Difference, HSD) to identify statistically significant differences between treatments (SPSS Statistics v.28).

#### 2.12.2. Principal Component Analysis

Principal component analysis (PCA) was applied to identify the variables responsible for most of the data variability caused by the different treatments. PCA was performed by using SPSS software, version 28 (IBM Corporation, New York, NY, USA).

## 3. Results and Discussion

### 3.1. Pre-Test Studies and Conditions Selection

There are few data available about the effect of US treatments on the inactivation of pathogenic microorganisms in egg products. For instance, US treatments at temperatures below 30 °C during long periods (up to 30 min) and with a working volume of 10–25 mL reduced *Salmonella* spp., *Listeria* spp., and *E. coli* by a maximum reduction of 2.3 log_10_ cycles [18,19,29]. Therefore, based on these results and aiming to sequentially combine US technology with TP and/or MP, a maximum treatment time of 5 min and a working volume of 50 mL was established. In fact, increasing the treatment volume decreased the energy applied per mL of liquid, thereby reducing the inactivation effect [18]. Despite this, in our study, we chose to work with a higher working volume compared to that in the studies cited above, as the US technology was to be applied in combination with other technologies.

The first US pre-tests conducted at 100% amplitude for 5 min produced a large amount of foam in LWE samples (Figure S1), and the following pre-tests revealed an amplitude of 50%, which corresponded to the first amplitude level with lower foam and was selected for further studies. However, under the defined conditions (50% amplitude/5 min), a “burning smell” was noticed in the egg samples (aroma linked with sonication treatment was also reported by some researchers [30]). To avoid this, in the present work, the treatment time was reduced to 1 min and the samples that were processed showed no signs of a burning smell.

Concerning MP pre-treatment, a treatment of 50 MPa for 5 min was previously explored in another study [31], showing no major changes in quality but a diminished impact on microbial inactivation, revealing that it could be a suitable condition for combination with other technologies.

In summary, a US pre-treatment of 50% amplitude during 1 min with a working volume of 50 mL, an MP pre-treatment at 50 MPa for 5 min, and a TP at commercial pasteurization temperature during a shorter time (60 °C/1.75 min) were chosen. Hence, the following combinations were examined: US–TP, MP–TP, US–MP, MP–US, US–MP–TP, MP–US–TP. They were compared with the respective commercial TP (60 °C/3.5 min [5,6]).

### 3.2. Microbial Analyses

**Figure 1:**
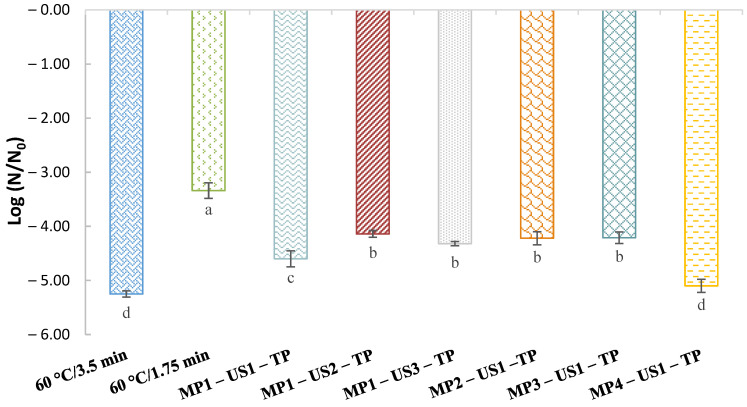
Inactivation of *Salmonella* Senftenberg 775/W (ATCC 43845), inoculated in liquid whole egg, by commercial thermal pasteurization (TP, 60 °C/3.5 min, 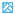); shorter TP (60 °C/1.75 min 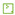), and moderate pressure (MP) and ultrasound (US), followed by a shorter TP: (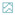) 50 MPa/5 min–50% amplitude/1 min–TP (MP1–US1–TP); (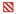) 50 MPa/5 min–50% amplitude/2 min–TP (MP1–US2–TP); (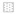) 50 MPa/5 min–50% amplitude/3 min–TP (MP1–US3–TP); (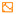) 90 MPa/5 min–50% amplitude/1 min–TP (MP2–US1–TP); (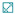) 125 MPa/5 min–50% amplitude/1 min–TP (MP3–US1–TP); (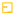) 160 MPa/5 min–50% amplitude/1 min–TP (MP4–US1–TP). N denotes the microbial load measured for each treatment and N_0_ denotes the initial microbial load (6.56–6.88 log CFU/mL, without any treatment). Different letters indicate significant differences (*p* < 0.05) between treatments.

The inactivation of *S.* Senftenberg 775/W was evaluated using MP, US, and TP, individually or sequentially combined, as shown in Table 1 and Figure 1. The lethal efficacy of the commercial TP against this *Salmonella* serovar was about 5.25 log_10_ cycles, but the shorter TP lowered only 3.34 log_10_ cycles. The MP (50 MPa/5 min) and US treatments (50% amplitude/1 min) (Treatments 1 and 6, Table 1), individually, reduced *S.* Senftenberg 775/W counts up to 0.26 log_10_ cycles, which was consistent with previously published findings on other *Salmonella* spp. [18,29] (Table 1). No significant differences were found when these two techniques were combined (US–MP (Treatment 12) or MP–US (Treatment 14), 0,37 log_10_ cycles, *p* ≥ 0.05), although other authors observed more promising results (up to 3 log_10_ cycles) after treating *S.* Enteritidis CB 919 Lux AB and *E. coli* K12 DH 5α using MP–US or US–MP combinations (US: 34.6–40 W/0.5–5 min/55 °C, MP: 138–300 MPa/3–15 min) [18,19].

In contrast, microbial inactivation was enhanced (up to 3.90 log_10_ cycles) when US or MP was applied before a shorter TP (US–TP and MP–TP, Treatments 5 and 9–11) (Table 1), compared to US and MP treatments, individually or combined with each other. In fact, a slightly higher inactivation was reported in our work (3.40–4.15 log_10_ cycles) than in the study of Monfort et al. [32], who inactivated up to 3.1 log_10_ cycles of *E. coli* K12 DH 5α (S. Enteritidis surrogate) applying a TP (52 °C/3.5 min and 55 °C/2 min) preceded by pressure (200–300 MPa/3–30 min) pre-treatments.

In addition, when US–MP and MP–US were also combined with a shorter TP (US–MP–TP (Treatment 13) and MP–US–TP), a significant improvement in *S.* Senftenberg 775/W reduction was found (up to 4.60 log_10_ cycles, *p* < 0.05) (Table 1 and Figure 1), showing that the pre-treatments combination order had a significant effect on inactivation. Likewise, as stated by other authors, using US as a second pre-treatment (MP–US–TP) resulted in greater inactivation, suggesting that a synergistic effect of US and TP acted on the injured survivors cells from the MP treatment [18]. As a comparison with commercial TP (5.25 log_10_ cycles), the MP–TP, US–TP, US–MP–TP, and MP–US–TP combinations resulted in a reduced inactivation (3.53–4.60 log_10_ cycles, *p* < 0.05), although the MP–US–TP combination resulted in a closer inactivation level.

A synergy was observed for MP–TP and MP–US–TP combinations, in which the combined effect was greater than the additive sum of each individual treatment, while US–TP and US–MP–TP combinations exhibited only additive effects. The MP–TP combination has been studied and been described in another work [31], whereas the US–TP combination does not appear to be a feasible better alternative, taking into account the microbiological results (inactivated 3.54 log_10_ cycles, which were far from the 5.25 log_10_ cycles of commercial TP) and the large increase in US treatment time required to achieve satisfactory possible microbial inactivation (which could result in quality deterioration, as observed in the pre-tests section). Therefore, only the optimization of the MP–US–TP combination was studied by increasing the US treatment time (1–3 min, without signs of a burning smell) or the MP intensity (50–160 MPa), as an attempt to replace commercial TP in achieving satisfactory microbial inactivation.

Initially, the US treatment time was increased (from 1 to 2 and 3 min), while the MP pre-treatment (50 MPa/5 min) was maintained (Table 1: Treatments 14–16, and Figure 1). As shown, individually or in combination with MP and shorter TP, the US treatment time increase, in general, did not increase the lethal effect on *S.* Senftenberg 775/W. Then, the US treatment was maintained at 50% amplitude for 1 min, and the pressure intensity was raised (50–160 MPa/5 min) (Table 1: Treatments 14 and 17–19, and Figure 1). Progressively increasing the MP intensity, applied individually or in combination with US and shorter TP, showed a *S*. Senftenberg 775/W inactivation increasing trend, but only with a significant impact at 160 MPa (from 4.60 to 5.10 log_10_ cycles). Furthermore, the MP–US–TP combination at 160 MPa/5 min–50% amplitude/1 min–60 °C/1.75 min (5.10 log_10_ cycles) obtained a lethal effect comparable to that of commercial TP (5.25 log_10_ cycles, *p* ≥ 0.05). Therefore, from a food safety point of view, the results indicate that the combination of 160 MPa/5 min–50% amplitude/1 min–60 °C/1.75 min could be an alternative to commercial TP and, therefore, main LWE quality properties were further assessed using this processing combination and they compared to commercial TP and each treatment individually.

In addition, in the case of HP technology, the effects on microorganisms can result from simultaneous damage to different cellular structures and functions [33], such as damage to the cell membrane and its structure, affecting their mechanisms of nutrient absorption and metabolite release; changes in proteins structure (denaturation, aggregation or gel formation) [34,35]; disruption of non-covalent bonds, mainly affecting tertiary and quaternary structures [36]; and multimeric disintegration ribosomes [37]. On the other hand, the cavitation effects are likely to be responsible for the impact of US technology. When bubbles collapse, this causes severe turbulence in the surrounding liquid, generating high-temperature (hotspots) and high-pressure zones and forming highly reactive free radicals [38,39,40]. These free radicals can act on the cell envelope, disrupt the cell walls, and, then, the cell dies [40,41]. In biomolecules, the US can induce changes in the protein structure (secondary and tertiary) due to the breakdown of hydrogen or Van der Walls bindings. In addition, the free radicals can react with amino acids residues involved in structure stability, substrate binding, or catalytic functions, and, consequently, change their biological activity [42]. Hence, when these technologies are applied at sublethal intensities, the resulting damage will likely affect the same structures, although to a lesser extent.

### 3.3. LWE Quality Properties Analyses

#### 3.3.1. Physicochemical Properties

Table 2 shows the physicochemical properties of LWE treated by MP followed by US before a shorter TP (MP–US–TP), compared with each treatment individually and commercial TP (60 °C/3.5 min).

The pH values varied from 7.9 (raw LWE) to 7.8 for the treated LWE samples, being the results for US-treated LWE (pH 7.8) in good agreement with those of O’Sullivan et al. [43]. On the contrary, the drop in pH found in MP-treated samples was not corroborated by previous published studies, which did not report changes in pH with pressure [44].

**Figure 2:**
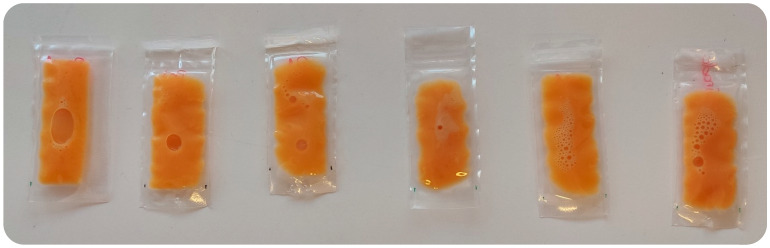
Images of whole egg samples packaged after each treatment. From left to right: raw whole egg, whole egg treated by commercial thermal pasteurization (60 °C/3.5 min), shorter thermal pasteurization (60 °C/1.75 min), moderate pressure (160 MPa/5 min), ultrasound (50% amplitude/1 min), and combined treatment (160 MPa/5 min–50% amplitude/1 min–60 °C/1.75 min).

The color of non-treated LWE was assessed and compared with the different treatments studied (Table 2 and Figure 2) and, overall, the treatments tended to increase the product lightness (L*, 44.0–43.5) and maintain redness (a*, 9.7–10,0), while a yellowness (b*, 9.9–11.6) decrement was found for MP-only and MP–US–TP treatments (*p* < 0.05). The color variations induced by the treatments (Δ*E* = 0.4–1.0) were in the range considered to be undetected by the naked eye (ΔE* < 3) (Figure 2) [45], with TP (shorter and commercial) exhibiting a less-pronounced effect (0.4–0.5). In fact, other authors have obtained comparable findings (ΔE* < 3) for egg products submitted to sequential combined treatments [22,23,32].

Concerning protein solubility (Table 2), a decrease of about 7–12% (100,0–87.9%) was found for LWE after shorter TP, commercial TP, and MP-only treatments compared to non-treated LWE (*p* < 0.05), with commercial TP causing a more significant loss (12%). Heat and MP can disrupt non-covalent bonds, resulting in protein unfolding by exposing hydrophobic groups buried inside, thereby promoting aggregation through hydrophobic interactions and reducing protein solubility [46]. Our results corroborate the findings of De Souza and Fernández and Yang et al. [47,48]. On the other hand, the treatments using US tended to improve protein solubility (compared to raw samples). Additionally, the MP–US–TP-treated (104,4%) LWE presented the highest protein solubility increment, up to 1.2-fold higher than the other samples (*p* < 0.05), pointing to the possibility that the negative effect of an MP pre-treatment (alone) may have been counteracted by the subsequent US treatment, as changes in protein structure are expected to be reversible within the studied pressure range [49]. The sonication could destroy the aggregated state of proteins, possibly exposing hydrophilic regions toward water molecules and, thereby, increasing protein solubility [15,50]. Thus, the following shorter TP had no impact on this property, unlike when it was applied alone.

According to Table 2, the apparent viscosity of raw LWE was 12.98 ± 1.03 mPa.s, which was consistent with the findings of other authors [32]. Compared to raw LWE, viscosity increased about 59% (20.6 mPa.s, *p* < 0.05) after both TP (shorter and commercial), and to a lesser extent with MP-only treatment (41%, 18.3 mPa.s, *p* < 0.05). These results are in line with the soluble protein decrement (Table 2) and probably occurred due to protein unfolding and aggregation [51], being supported by the findings of Monfort et al. and Souza and Fernández [32,47]. On the contrary, US-only (8.6 mPa.s) and MP–US–TP-treated (9.1 mPa.s) LWE exhibited a lower viscosity than that of non-treated LWE (13.0 mPa.s, 30–34%, *p* < 0.05), which was accompanied by an increase in protein solubility. Indeed, a previous study of Sheng et al. [15] suggested that mechanical energy and the cavitation effect decreased the aggregation degree between the proteins’ molecules, thereby lowering the viscosity of sonicated egg white (EW) [15]. Moreover, the samples treated by MP–US–TP had a viscosity 2.3-fold lower than MP- and shorter TP-treated LWE (*p* < 0.05), although no significant difference was identified when compared to US-treated LWE (*p* ≥ 0.05), suggesting that the effect observed for combined treatment can probably be attributed to the sonication effects. In this case, the initial viscosity increase induced by MP treatment appears to have been reversed (at pressures below 300 MPa, pressure-induced changes on protein structure are expected to be reversible) during sonication. Thus, further protein aggregation caused by thermal treatments occurred to a much lesser extent than when shorter TP was applied alone [15,49,52]. Analogous results were reported by Ashokkumar et al. [52], who found a reduction in viscosity of whey protein concentrate submitted to a pre-heat treatment (80 °C/1 min) followed by sonication (for less than 5 min) and by a post-heat treatment (85 °C/20 min). Furthermore, a 2.3-fold less viscous LWE was obtained with the combined treatment (9.1 mPa.s) than with commercial TP (20.6 mPa.s), which might be advantageous for industrial LWE-processing (pumping, mixing, and so on).

#### 3.3.2. Thermal Properties

The thermal properties of LWE were investigated at the rate of 10 °C/min across the temperature range of 30 to 120 °C (Table 2). According to the findings of other authors [26] only one major endotherm was identified in LWE samples, and the peak was visible in all the treatments. The peak denaturation temperature of raw LWE did not change significantly after treatments, with the exception of the MP-only treatment (83.83–85.2 °C, *p* ≥ 0.05), presumably indicating a similar denaturation process for this protein fraction and, hence, no major structural changes caused by the treatments. The Δ*H* of both TP-, US- and MP–US–TP-treated LWE did not vary significantly from that of raw LWE (4.3–6.1 J/g, *p* ≥ 0.05), but TP and US treatments exhibited a decreasing trend. However, it seems that US and shorter TP reversed the effects of MP on LWE treated with MP–US–TP, as reported above. Additionally, for the MP-only treatment, there was a higher peak denaturation temperature (87.5 °C) and a lower Δ*H* (3.94 J/g), compared to raw LWE (*p* < 0.05), possibly indicating a partial loss of protein structure in this sample [53]. Indeed, these data were comparable with previous investigations that reported a decrease in these parameters at pressures as low as 100 MPa [13,26].

#### 3.3.3. Emulsifying Properties

In general, all treatments reduced the emulsifying properties of raw LWE (EAI: 65.8 m^2^/g; ESI: 1.3 min, *p* < 0.05), with the exception of MP-only treatment, which had no effect on the EAI (67.1 m^2^/g, *p* ≥ 0.05) but increased the ESI (1.7 min, *p* < 0.05) (Table 2). For MP-treated LWE, the observed rise in ESI may be related to the moderate protein unfolding [13], consistent with the protein solubility results. The lack of effect caused by MP on EAI was corroborated by the study of Khan et al. [54] when they subjected a sweet potato protein-guar gum model to an MP treatment (200 MPa for 20 min at 25 °C). In addition, the TP (shorter and commercial) significantly reduced the emulsifying properties (EAI: 40,8–44.2 m^2^/g and ESI: 0.6–1.0 min, *p* < 0.05), possibly as a result of proteins unfolding and aggregation, thereby hindering protein adsorption at the interfaces [55]. Therefore, when fewer aggregates adsorb at the interface, the emulsion stability decreases, as this property is related to the interfacial area that can be coated by proteins [56]. In contrast to our observations, Le Denmat et al. [55], found that egg yolk’s (EY’s) emulsifying properties were unaffected at temperatures below 69 °C.

An even higher decrement in emulsifying properties was found for LWE treated with US-only (EAI: 23.4 m^2^/g; ESI: 0.6 min) and MP–US–TP (EAI: 24.8 m^2^/g; ESI: 0.7 min) treatments (43–65%), which may be due to changes in protein molecular flexibility [57]. Our findings were comparable with those of Zhou et al. [57], who stated that a sonication treatment lowered the EAI of glycinin while increasing the protein solubility. In contrast, several authors observed a positive effect of sonication on the emulsifying properties of EW and EY solutions [50,58]. Considering the combined treatment, LWE presented similar emulsifying properties to those of US-treated LWE (*p* ≥ 0.05), indicating that the impact of MP pre-treatment was countered by the subsequent sonication, as mentioned above, and the following treatment at 60 °C had no significant effect. In comparison to commercial TP (EAI: 44.2 m^2^/g; ESI: 0.6 min), lower emulsifying properties were obtained for MP–US–TP-treated LWE (EAI: 24.8 m^2^/g; ESI: 0.7 min).

#### 3.3.4. Secondary Lipid Oxidation

The secondary lipid oxidation (TBARS, expressed as MDA content) was evaluated as an estimation of the extent of lipid oxidation caused by processing. The measured TBARS values for raw LWE were 13.3 ± 0.6 µg MDA/100 g whole egg (Table 2), similar to what was described by other authors [59]. TP and US treatments alone did not cause any significant impact on MDA content (12.9–13.3 µg MDA/100 g whole egg, *p* > 0.05), in comparison with non-treated LWE, similar to what was observed by other authors [45,60]. Compared to the non-treated LWE samples or those treated by commercial TP, the MP treatment alone caused a significant decrease in MDA content (about 20% reduction; 10.7 µg MDA/100 g; *p* < 0.05), which may be due to the degradation or production of tertiary lipid oxidation products that are not detected by the TBARs method [61,62]. However, the combined treatment caused a slight increase (from 13,1 to 13,8 µg MDA/100 g, *p* < 0.05) (Table 2), which was probably associated with a more pronounced contribution of the US treatment, as previously verified for protein solubility and viscosity. Literature data on the effect of pressure treatments on lipid oxidation can be considered somewhat controversial. Some researchers found no significant changes on the TBARS values of pork as a result of MP treatment (up to 200 MPa) [63], although, other authors reported an increase in TBARS values of pressure-treated coho salmon (200 MPa/30 sec) [64] or even a decrease in sliced cooked ham treated at 100 MPa for 8 h at 30 °C [62].

#### 3.3.5. Volatile Profile

The compositions of LWE samples in regard to identified volatile compounds are listed in Table 3. In order to prevent the eventual formation of new volatiles induced by the temperature used in the extraction procedure, the volatiles were extracted at 45 °C, a lower temperature than that used for LWE TP (60 °C). A total of 14 volatiles were found using HS-SPME analysis, of which six were identified.

In general, the total volatiles content of raw LWE (168.73 µg/100 g) decreased with US-only (38.13 µg/100 g), MP-only (13.43 µg/100 g), and MP–US–TP (23.80 µg/100 g) treatments (*p* < 0.05), with a more pronounced impact of the MP-only treatment (−92%), while neither TP (shorter or commercial) changed the total volatiles content (*p* ≥ 0.05). The volatile profile of LWE consisted essentially of hydrocarbons, with heptane and hexane being the most abundant, as previously observed in eggs [65]. MP- (6.96 µg/100 g) and US-treated (5.03 µg/100 g) LWE contained a considerably lower content of these compounds (3.5–8.7-fold) compared to raw (28.02 µg/100 g) and both TP-treated (33.64–35.14 µg/100 g) LWE (*p* < 0.05), whereas in MP–US–TP-treated samples, only hexane (2.52 µg/100 g) content significantly decreased. The other identified hydrocarbon compounds were 2-methyl pentane, 3-methyl pentane, and decahydro-2-methylnaphthalene, the latter being already reported in a previous study [66]. In general, for these compounds, no significant differences were found between raw and treated samples (*p* ≥ 0.05). In addition, a 1.9-fold increase in 2-methyl pentane was detected in LWE treated by MP–US–TP (0.33 µg/100 g) in comparison to the commercial TP-treated (0.18 µg/100 g) LWE, although the former presented a lower content of 3-methyl pentane and decahydro-2-methylnaphthalene (1.5–1.9-fold lower) (*p* < 0.05). Therefore, the sum of hydrocarbons in US-, MP- and MP–US–TP-treated LWE was up to 80% lower (*p* < 0.05) than in the other samples. However, owing to the high-odor threshold values of hydrocarbons, it is unlikely that significant differences in their contents result in important changes in aroma perception [67]. Toluene was the compound found in the highest amount in raw and both TP-treated LWE (113.08–133.22 µg/100 g, *p* ≥ 0.05), being previously identified by other authors [28,65]. A decrease of 97% was found in MP-(3.60 µg/100 g) and MP–US–TP-treated (4.33 µg/100 g) LWE compared to raw and both TP-treated samples (*p* < 0.05); however, this decrement was much higher than that produced by US-only treatment (29.71 µg/100 g, decrease about 78%, *p* < 0.05). The obtained findings for MP-treated samples were consistent with the findings of Contador et al. [68], who noticed a reduction in aliphatic hydrocarbons and toluene after applying a pressure treatment for 6 min. Otherwise, US treatment enhanced hydrocarbon content in spiced beef using more severe treatment conditions [67], and the occurrence of pyrolysis when cavitation bubbles collapsed was a possible mechanism for the generation and degradation of volatile compounds [69].

In comparison with non-treated LWE, compounds such as aldehydes, alcohols, and ketones, which are mainly found in cooked eggs and egg samples submitted to extractions at higher temperatures and longer times [63,70], were not detected in treated samples because the treatments applied were insufficient to induce their production. Consequently, the results demonstrate that the LWE volatile profile was significantly more affected by MP–US–TP treatment (23.80 µg/100 g) than by commercial TP (166.38 µg/100 g), with the former being characterized by the low compounds content.

#### 3.3.6. Principal Component Analysis

**Figure 3:**
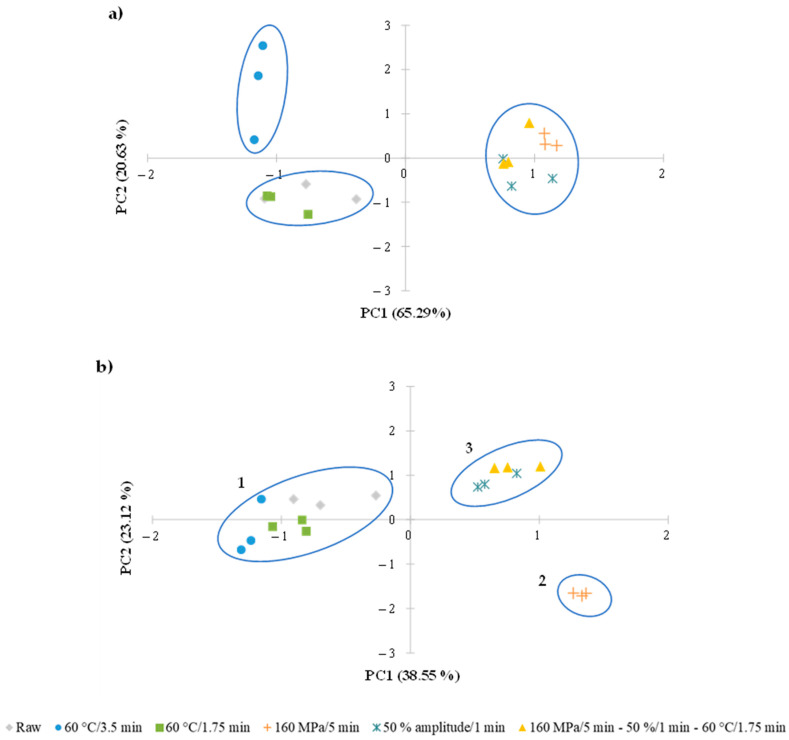
Principal component analysis (PCA) score plot for (**a**) volatile compounds and (**b**) thermal, physicochemical and functional properties plus volatile compounds of raw and treated whole egg. The principal components (PC) explain 85.92% (**a**) and 61.67% (**b**) of the total variance of the data.

The measured parameters for the treated LWE samples were submitted to a PCA in order to obtain further information and to determine the variables responsible for the majority of the data variability induced by the different treatments. Regarding volatile compounds’ composition, as seen in Figure 3a, the first two principal components (PC1 and PC2) accounted for 65.29 and 20.63% of the total variance, respectively. The findings revealed three well-defined groups, which corresponded to (1) commercial TP-treated LWE, (2) raw and shorter TP-treated LWE, and (3) US-, MP- and MP–US–TP-treated samples. Group 1 was mainly influenced by the 3-methyl pentane and decahydro-2-methylnaphthalene content, whereas group 2 was distinguished by the highest amount of toluene and hydrocarbons compounds (Table S1). Additionally, group 3 was characterized by the lowest compounds content.

PCA was also performed, taking into consideration the other measured properties. Figure 3b shows the factorial coordinate diagram representing the score plots of pH, color, protein solubility, viscosity, thermal properties, emulsifying properties, secondary lipid oxidation, and volatile compounds. As shown, the first two PCs explained 38.55 and 23.12% of the total variance, respectively, observed for the raw and treated LWE samples, distinguishing three distinct groups of points corresponding to: (1) raw, shorter TP- and commercial TP-treated LWE; (2) MP-treated LWE; and (3) US- and MP–US–TP-treated LWE. The raw and both TP-treated samples were distinguished from the other samples by changes in pH, ∆*H*, TBARS, and protein solubility (Table S2), while group 2 was clearly distinguished by the lowest pH, TBARS, and ∆*H*. Furthermore, the samples submitted to US-only and MP–US–TP treatments were distinguished from the others mainly by the highest protein solubility and the 2-methyl pentane content.

Therefore, the results show that the TP caused less changes (negative) in LWE properties than the other treatments evaluated, whereas the MP-only treated samples were the most distinct. Consequently, these results indicate that US-only, MP-only or MP–US–TP treatments induced overall more pronounced changes (negative) in raw LWE properties than thermal treatments.

## 4. Conclusions

The application of MP (160 MPa/5 min) followed by US (50% amplitude/1 min) prior to a shorter TP (60 °C/1.75 min) could be an effective method to pasteurize LWE. In comparison to LWE commercial TP, the MP–US–TP combination improved protein solubility and lowered viscosity, while decreasing the volatile compounds’ content and emulsifying capacity. Overall, although the MP–US–TP treatment provides a similar level of safety as commercial TP, the final product obtained with commercial TP was more similar to non-treated LWE (quality properties) than the MP–US–TP-treated LWE.

By adding pre-treatment technologies upstream, this strategy reduces the severity of a single technique applied alone, while facilitating its industrial implementation. Nevertheless, its limitations are mostly associated with the difficulty of US scale-up and the large initial investment in HP equipment. The promising results obtained in this study suggest that the sequential combination of MP and US followed by a shorter TP may be a possible alternative to commercial LWE TP. However, additional research is required to improve our understanding of the effect of this treatment, including its effect on microorganisms during storage, gelling properties of LWE heat-induced gels, primary and tertiary lipid oxidation, total carotenoid content, and the sensorial characteristics of egg products derived from treated LWE.

## Figures and Tables

**Table 1:** Log_10_ cycles reductions in the population of *Salmonella* Senftenberg 775/W (ATCC 43845) in liquid whole egg (with an initial microbial load (N_0_) of 6.55–6.98 log CFU/mL) treated by moderate pressure (MP, 50–160 MPa/5 min), ultrasound (US, 50% amplitude/1–3 min), MP followed by a shorter thermal pasteurization (TP, 60 °C/1.75 min) (MP–TP), US followed by a shorter TP (US–TP), US followed by MP and/or followed by a shorter TP (US–MP and US–MP–TP), or MP followed by US (MP–US). Different letters indicate significant differences (*p* < 0.05) between treatments.

Treatments		*Salmonella* Senftenberg 775/W
		MP
1	50 MPa/5 min	0.05 ± 0.06 ^a^
2	90 MPa/5 min	0.11 ± 0.11 ^ab^
3	125 MPa/5 min	0.16 ± 0.09 ^abc^
4	160 MPa/5 min	0.67 ± 0.07 ^g^
		MP–TP
5	50 MPa/5 min–60 °C/1.75 min	3.90 ± 0.17 ^j^
		US
6	50% amplitude/1 min	0.26 ± 0.17 ^bcde^
7	50% amplitude/2 min	0.25 ± 0.05 ^bcde^
8	50% amplitude/3 min	0.38 ± 0.02 ^ef^
		US–TP
9	50% amplitude/1 min–60 °C/1.75 min	3.54 ± 0.15 ^i^
10	50% amplitude/2 min–60 °C/1.75 min	3.40 ± 0.05 ^i^
11	50% amplitude/3 min–60 °C/1.75 min	4.15 ± 0.06 ^k^
		US–MP
12	50% amplitude/1 min–50 MPa/5 min	0.31 ± 0.15 ^cde^
		US–MP–TP
13	50% amplitude/1 min–50 MPa/5 min–60 °C/1.75 min	3.53 ± 0.15 ^i^
		MP–US
14	50 MPa/5 min–50% amplitude/1 min	0.37 ± 0.14 ^def^
15	50 MPa/5 min–50% amplitude/2 min	0.35 ± 0.03 ^def^
16	50 MPa/5 min–50% amplitude/3 min	0.50 ± 0.02 ^fg^
17	90 MPa/5 min–50% amplitude/1 min	0.20 ± 0.06 ^abcd^
18	125 MPa/5 min–50% amplitude/1 min	0.30 ± 0.07 ^cde^
19	160 MPa/5 min–50% amplitude/1 min	0.87 ± 0.08 ^h^

**Table 2:** Physicochemical, thermal, and functional properties and lipid oxidation of raw and treated liquid whole egg (mean ± standard deviation). Different letters along each row denote significant differences (*p* < 0.05) between processing conditions.

Properties	Raw	Thermal Pasteurization		Ultrasound	**Moderate Pressure**	**Combined Process (MP–US–TP) ^1^**
		60 °C/3.5 min	60 °C/1.75 min	50%/1 min	160 MPa/5 min	160 MPa/5 min–50%/1 min–60 °C/1.75 min
pH	7.9 ± 0.0 ^a^	7.9 ± 0.0 ^ab^	7.9 ± 0.00 ^b^	7.8 ± 0.0 ^c^	7.8 ± 0.0 ^d^	7.8 ± 0.0 ^c^
L*	44.0 ± 0.7 ^bc^	44.7 ± 0.12 ^a^	44.3 ± 0.7 ^ab^	44.7 ± 0.2 ^a^	44.1 ± 0.2 ^b^	43.5 ± 0.7 ^c^
a*	9.8 ± 0.5 ^a^	10.0 ± 0.1 ^a^	9.7 ± 0.4 ^a^	9.9 ± 0.0 ^a^	10.0 ± 0.0 ^a^	9.8 ± 0.4 ^a^
b*	11.1 ± 0.9 ^ab^	11.3 ± 0.3 ^ab^	11.0 ± 0.7 ^bc^	11.6 ± 0.1 ^a^	10.5 ± 0.2 ^c^	9.9 ± 0.7 ^d^
∆*E*	-	0.5 ± 0.2 ^c^	0.4 ± 0.1 ^c^	1.0 ± 0.1 ^a^	0.8 ± 0.1 ^b^	0.8 ± 0.1 ^b^
Protein solubility (%)	100.0 ± 3.4 ^b^	87.9 ± 2.9 ^d^	92.6 ± 3.2 ^c^	103.2 ± 2.6 ^ab^	90.8 ± 2.0 ^cd^	104.4 ± 3.9 ^a^
Viscosity (mPa.s, at shear rate of 50 s^−1^)	13.0 ± 1.0 ^c^	20.6 ± 1.3 ^a^	20.5 ± 1.4 ^a^	8.6 ± 0.1 ^d^	18.3 ± 0.5 ^b^	9.1 ± 0.1 ^d^
T_onset_ (°C)	75.9 ± 0.1 ^a^	77.9 ± 0.1 ^a^	77.6 ± 2.2 ^a^	78.3 ± 0.5 ^a^	80.0 ± 1.6 ^a^	77.8 ± 1.2 ^a^
T_peak_ (°C)	84.2 ± 0.6 ^b^	83.8 ± 1.0 ^b^	84.2 ± 0.1 ^b^	84.9 ± 0.1 ^b^	87.5 ± 0.1 ^a^	85.2 ± 0.4 ^b^
∆*H* (J/g)	6.7 ± 0.8 ^a^	4.4 ± 0.4 ^ab^	4.7 ± 1.0 ^ab^	4.3 ± 0.3 ^ab^	3.94 ± 0.9 ^b^	6.1 ± 0.4 ^ab^
Emulsifying Activity Index (m^2^/g)	65.8 ± 4.2 ^a^	44.2 ± 4.8 ^b^	40.8 ± 1.0 ^b^	23.4 ± 0.6 ^c^	67.1 ± 0.8 ^a^	24.8 ± 0.6 ^c^
Emulsifying Stability Index (min)	1.3 ± 0.3 ^b^	0.6 ± 0.0 ^d^	1.0 ± 0.2 ^c^	0.6 ± 0.1 ^d^	1.7 ± 0.1 ^a^	0.7 ± 0.1 ^d^
Lipid Oxidation (TBARS, µg/100 g whole egg)	13.3 ± 0.6 ^ac^	13.1 ± 0.5 ^a^	12.9 ± 0.6 ^a^	13.3 ± 0.4 ^ac^	10.7 ± 0.3 ^b^	13.8 ± 0.3 ^c^

^1^ MP: moderate pressure; US: ultrasound; TP: thermal pasteurization.

**Table 3:** Volatile profile of raw and treated whole egg was evaluated by headspace solid-phase microextraction and analyzed by gas chromatography–mass spectrometry, expressed in µg internal standard equivalents/100 g whole egg (mean ± standard deviation). Different letters along the same line indicate significant differences (*p* < 0.05) between conditions.

Volatile Compounds	RT (min)	Liquid Whole Egg (µg/100 g Whole Egg) ^1,2^					
		Raw	60 °C/3.5 min	60 °C/1.75 min	50%/1 min	160 MPa/5 min	160 MPa/5 min–50%/1 min–60 °C/1.75 min
2-methyl pentane	2.962	0.20 ± 0.06 ^abc^	0.18 ± 0.04 ^bc^	0.16 ± 0.02 ^c^	0.23 ± 0.09 ^abc^	0.32 ± 0.05 ^ab^	0.33 ± 0.04 ^a^
3-methyl pentane	3.088	0.24 ± 0.02 ^b^	0.47 ± 0.06 ^a^	0.27 ± 0.01 ^b^	0.30 ± 0.06 ^b^	0.36 ± 0.04 ^ab^	0.32 ± 0.04 ^b^
Hexane	3.242	9.52 ± 1.45 ^a^	9.38 ± 2.85 ^a^	12.32 ± 0.61 ^a^	2.90 ± 0.33 ^b^	2.72 ± 0.65 ^b^	2.52 ± 0.25 ^b^
Heptane	5.002	18.50 ± 8.80 ^a^	24.26 ± 3.28 ^a^	22.82 ± 0.54 ^a^	2.13 ± 0.44 ^b^	4.24 ± 1.11 ^b^	12.91 ± 4.51 ^ab^
Decahydro-2-methylnaphthalene	22.273	0.11 ± 0.01 ^b^	0.19 ± 0.05 ^a^	0.09 ± 0.02 ^b^	0.08 ± 0.00 ^b^	0.09 ± 0.02 ^b^	0.10 ± 0.02 ^b^
∑ Hydrocarbons	-	35.51 ± 9.78 ^a^	41.06 ± 4.27 ^a^	42.63 ± 0.59 ^a^	8.42 ± 0.66 ^b^	9.83 ± 1.66 ^b^	19.47 ± 4.31 ^b^
Toluene	7.498	133.22 ± 7.28 ^a^	125.31 ± 10.51 ^a^	113.08 ± 27.91 ^a^	29.71 ± 1.15 ^b^	3.60 ± 0.11 ^b^	4.33 ± 0.22 ^b^
∑ Total	-	168.73 ± 8.89 ^a^	166.38 ± 6.34 ^a^	155.71 ± 28.34 ^a^	38.13 ± 1.53 ^b^	13.43 ± 1.77 ^b^	23.80 ± 4.50 ^b^

^1^ Values are from semi-quantification using cyclohexanone as internal standard; ^2^ Detection limit is 0.01 µg/100 g whole egg.

## Data Availability

The data presented in this study are available on request from the corresponding author.

## Supplementary Materials

The following supporting information can be downloaded at: https://www.mdpi.com/article/10.3390/foods12132459/s1, Figure S1: Samples of whole egg treated by ultrasound at 100% amplitude during 5 minutes.; Table S1: Loadings of the variables in the first two principal component (PC) analysis of volatile compounds in liquid whole egg samples; Table S2: Loadings of the variables in the first two principal component (PC) analysis of thermal, physicochemical and functional properties and lipid oxidation plus volatile compounds in liquid whole egg samples.
