# Supplementary material for: Effects of Sequential Combination of Moderate Pressure and Ultrasound on Subsequent Thermal Pasteurization of Liquid Whole Egg
Source: Foods. 2023 Jun 23;12(13):2459. doi: 10.3390/foods12132459 (PMC10340185; doi:10.3390/foods12132459)
Supplement: Supplementary file 1 [file foods-12-02459-s001.zip › foods-2147501-supplementary.pdf]

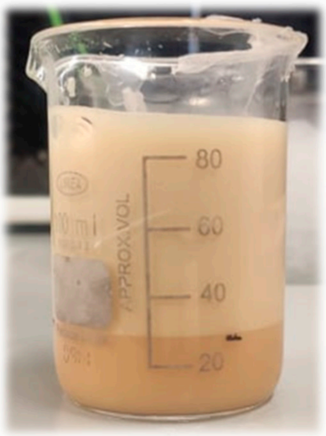

Figure S1. Samples of whole egg treated by ultrasound at 100 % amplitude during 5 minutes.

Table S1. Loadings of the variables in the first two principal component (PC) analysis of volatile compounds in liquid whole egg samples.

Compounds	Principal Components	
	PC 1	PC 2
2-methyl pentane	0.732	0.211
3-methyl pentane	-0.092	0.935
Hexane	-0.929	-0.246
Heptane	-0.898	0.056
Decahydro-2-methylnaphthalene	-0.491	0.812
Toluene	-0.949	-0.054
Hydrocarbons compounds	-0.959	-0.049

Table S2. Loadings of the variables in the first two principal component (PC) analysis of thermal, physicochemical and functional properties and lipid oxidation plus volatile compounds in liquid whole egg samples.

Compounds	Principal Components	
	**PC 1**	**PC 2**
pH	-0.809	0.558
L*	-0.326	-0.156
a*	-0.008	-0.207
b*	-0.446	-0.029
Protein solubility	0.398	0.868
Lipid oxidation	-0.238	0.931
Emulsifying activity index	-0.083	-0.684
Emulsifying stability index	0.371	-0.606
Viscosity	-0.518	-0.794
T_{peak}	0.807	-0.459
ΔH	-0.144	0.621
2-methyl pentane	0.748	0.011
3-methyl pentane	-0.116	-0.481
Hexane	-0.902	-0.114
Heptane	-0.876	-0.029
Decahydro-2-methylnaphthalene	-0.489	-0.281
Toluene	-0.936	-0.056
Hydrocarbons compounds	-0.936	-0.047
